# A Retrospective Study on Bovine Fascioliasis in Veterinary Regional Laboratories in Peninsular Malaysia

**DOI:** 10.1155/2019/7903682

**Published:** 2019-07-01

**Authors:** J. Nur Alia Diyana, I. H. Lokman, S. H. Nur Fazila, H. Latiffah, E. B. Ibitoye, H. Noor Hazfalinda, P. Chandrawathani, Kamaludeen Juriah, M. I. Nur Mahiza

**Affiliations:** ^1^Faculty of Veterinary Medicine, Universiti Putra Malaysia, 43400, Serdang, Selangor, Malaysia; ^2^Faculty of Veterinary Medicine, Usmanu Danfodiyo University, P.M.B 2346, Sokoto, Nigeria; ^3^Faculty of Health Sciences, Universiti Kebangsaan Malaysia, 43600 UKM Bangi, Selangor, Malaysia; ^4^Research and Innovation, Department of Veterinary Services, 62624 Putrajaya, Malaysia; ^5^Faculty of Agriculture and Food, Universiti Putra Malaysia Bintulu Sarawak, 97008 Sarawak, Malaysia

## Abstract

This is a retrospective study on bovine fascioliasis infection on cattle in Peninsular Malaysia, spanning from January 2007 to December 2017. Cattle were diagnosed with* Fasciola* based on the fecal examination and the results were reported to the Veterinary Regional Laboratories in Peninsular Malaysia. These records were analysed for the occurrence of bovine fascioliasis within that 11-year period. Records of annual diagnostic cases from five major Veterinary Regional Laboratories were examined: Bukit Tengah, Pulau Pinang (north); Kuantan, Pahang (east); Johor Bahru, Johor (south); Sepang, Selangor (west); and Kota Bharu, Kelantan (east). A positive fascioliasis infestation was calculated based on a number of positively infected cattle with* Fasciola* from a number of cattle examined. A total of 1988 cattle were examined during this period and 35 (1.76%) cattle were reported to be positive for bovine fascioliasis. Parasite infection was the highest at Bukit Tengah region (5.55%) where 19 cases were positive from 342 reported cases while, at Kuantan (4.96%), 15 positive cases were reported, unlike Johor Bahru (0.09%), with only 1 positive case from 1136 cattle examined. Sepang and Kelantan had no positive cases. These results showed that bovine fascioliasis was generally prevalent in the northern and southeast parts of the Peninsular Malaysia; however, there was no significant relationship between the region and the occurrence of fascioliasis.

## 1. Introduction

**Figure 1:**
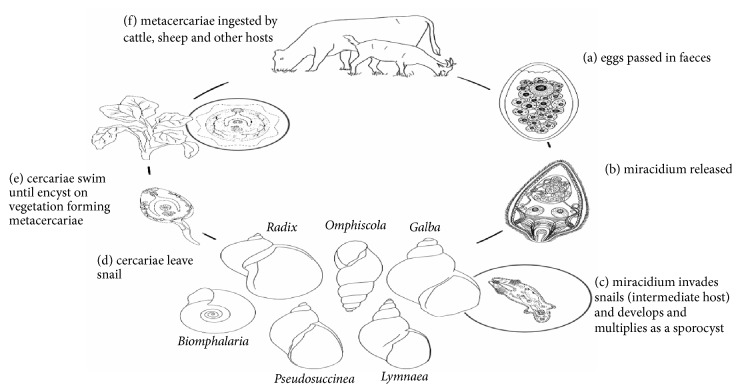
The life cycle of* Fasciola* in ruminants.

Livestock is one of the rapidly evolving sectors in agriculture, offering potential opportunities for economic growth and alleviation of poverty among rural dwellers, by generating market opportunities for the poor livestock-dependent and enhancing food security and nutrition [1]. However, parasitic diseases, such as fascioliasis, are considered major obstacles for the efficient production and maintenance of health as well as food safety of animal origin. They can cause significant economic loss in countries with livestock industry as an important segment of the agricultural products [2, 3]. Fascioliasis is a parasitic disease caused by liver flukes of the* Fasciola* genus and is of importance for both farm animals (especially ruminants) and humans [4, 5]. Fascioliasis infections have increased worldwide in the last decade and it is reported that 2.5 million people in 61 countries were infected by these parasites in addition to over 180 million people being at risk [6]. This disease has been considered a worldwide problem as it was reported in both developed and developing countries [4]. Animal fascioliasis continued to evolve due to unregulated movements of infected animals from region to region, where* Fasciola* spp. had been endemic for several years. Besides, moisture and the optimal temperature above 10°C found in these regions are essential aspects for the growth of miracidia, the reproduction of the snails (the intermediate host-*Lymnaea auricularia rubiginosa*), and larval development [2, 7]. Cattle were most likely infected with fascioliasis when they graze on grasses near the lake or river where metacercariae are attached on the grass [8]. In India, the prevalence of this disease is high in areas surrounding dams or large ponds where* Lymnaea auricularia rubiginosa*, the intermediate host of* F. gigantica*, is found [9]. The snail acts as the main factor for development of miracidia into metacercariae which is ingested by the cattle [10] [see Figure 1].

Economic losses from fascioliasis in ruminants (goats, sheep, cattle, and buffaloes) are usually due to a drop in livestock production, growth reduction, liver condemnation at slaughter, reduction in draught power, and high usage of anthelmintic [11]. However, the estimation of production and economic loss due to fascioliasis at national and regional level is limited due to lack of accurate estimation of fascioliasis prevalence. Hence, it is essential to have information on the status of parasitic diseases with regard to its magnitude of occurrence, negative production, and economic impact from different parts of the country to establish appropriate strategy for prevention and control of this disease. Therefore, the objective of this study was to determine the occurrence of bovine fascioliasis over time, diagnosed in five major regional veterinary laboratories in Peninsular Malaysia.

## 2. Materials and Methods

This study was carried out using samples submitted to the Regional Veterinary Laboratories (RVL) located in Johor Bahru, Kuantan, Kota Bahru, Sepang, and Bukit Tengah in Peninsular Malaysia. Each RVL receives samples from different states as shown in Table 1. There are several regional laboratories that serve the entire Malaysia and are concentrated in regions of high number of animals. The retrospective data were collected for the period of eleven years from January 2007 to December 2017.

Each laboratory database includes information of the source of the sample, data of submission date, and breed of animals. Records were examined on annual basis with regard to cases of fascioliasis reported in cattle. The occurrence of fascioliasis was calculated as the proportion of positive samples out of the samples that were submitted. The proportion of samples that tested positive by region or by year was computed in a similar way. The significance of association between the occurrence of fascioliasis and a region was evaluated using the logistic regression analysis and quantified by computing the odds ratio. Data were entered, validated, and calculated in Microsoft® Excel 2007 spreadsheet.

## 3. Results and Discussion

A total of 1988 fecal samples were examined during the period of eleven years and 35 (1.76%) of these samples tested positive for fascioliasis. Occurrence of bovine fascioliasis was the highest in Bukit Tengah RVL with 5.55% (19/342), followed by Kuantan RVL, 4.96% (15/302). In Johor Bahru RVL, 0.09% (1/1136) positive cases were reported, while both Sepang and Kota Bahru RVLs did not report any positive samples during the study period (Table 2).

**Figure 2:**
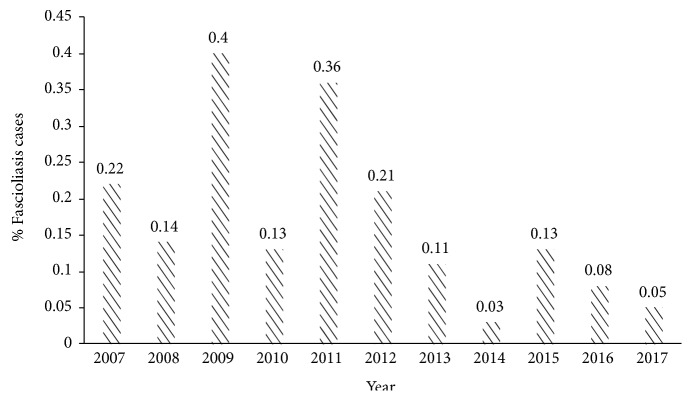
Annual trends of fascioliasis cases in five Regional Veterinary Laboratories in Peninsular Malaysia.

**Figure 3:**
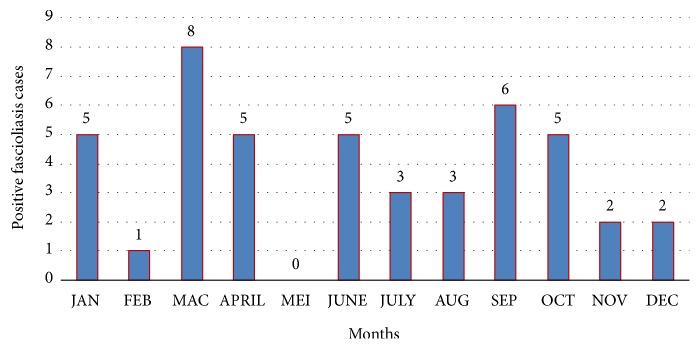
Monthly trends of fascioliasis cases in five Regional Veterinary Laboratories in Peninsular Malaysia.

For the annual trend of bovine fascioliasis, the highest occurrence was reported in 2009 (0.40%), while the lowest proportion of fascioliasis-positive samples was observed in 2014 (0.03%), as shown in Figure 2. On monthly trend of fascioliasis, most numbers of cases were reported in March (eight cases) while the least numbers of bovine fascioliasis cases were reported in May (zero cases) as in Figure 3.

This study reported present status of* Fasciola* infections in cattle diagnosed in the selected five Regional Veterinary Laboratories from 2007 to 2017. The results of this study suggested that bovine fascioliasis occurred in the study area, however in a less severe manner with a prevalence of 1.76% (35/1988). This result was lower than a prevalence of 3.68% (385/10462) reported in the liver of cattle slaughtered in 2012–2013 at an abattoir in Kashan region, Center Iran [12], and 28.6% reported in cattle in Southwest China [13]. In contrast, the prevalence reported in this current study was higher than reported cases in Northeastern Iran, which was 0.71% (35/4933) [14]. These variations in the prevalence of fascioliasis might be attributed to the differences in the climate and ecological settings such as rainfall, seasons, altitude, temperature, origin, and types of animals studied as well as differences in the host immune response to this parasite and the livestock management system. In addition, differences in study design may have also contributed to this varied prevalence. Retrospective data were generated in this current study, while others [12] used a cross-sectional approach.

The current study noted that the highest prevalence of bovine fascioliasis, 5.55% (19/342), was obtained from Bukit Tengah RVL. A reason for this might be that Bukit Tengah is located in the northern part of the Peninsular Malaysia, which receives samples from Kedah, Penang, and Perlis. The northern parts of Malaysia were known as the rice bowl of the country [15, 16], a location favouring the thriving of snail that acts as the intermediate host of fascioliasis. Therefore, it is expected that the numbers of intermediate host were high in this area and the fascioliasis cases were higher as well [17]. This present result agreed with researchers in Vietnam [17], in that high population of intermediate host snails indicated higher risk of fascioliasis. Furthermore, a research conducted in Indonesia [18] also agreed with the current study that the prevalence of* F. gigantica* infection was high in cattle and buffaloes raised around rice-producing areas where these intermediate host snails thrive.

Monthly trends showed that the highest occurrence for bovine fascioliasis was in March, with eight cases, while no cases of bovine fascioliasis were reported in May. This may be due to the variation in rainfall and number of snail population. A study on the life cycle of* F. gigantica* in Malawi indicated that the cercariae are released from July to October and cattle are thus exposed to a higher level of infection from August onwards [19]. This probably explains the pattern of distribution noted in this present study.

## 4. Conclusion

It can be concluded that the prevalence of* Fasciola* infections among cattle in the selected areas is mild, but cattle examined in the Bukit Tengah RVL in the northern part of Malaysia are at higher risk. Nevertheless, herders and policy makers ought to be abreast of this so that practices and policies that will help in maintenance of animals' health are instituted.

## Figures and Tables

**Table 1:** Sources of samples to the five Regional Veterinary Laboratories in Peninsular Malaysia.

	Regional Veterinary Laboratories				
	Kuantan	Bukit Tengah	Kota Bahru	Johor Bahru	Sepang
States	Pahang	Perlis	Terengganu	Johor	Selangor
	Negeri Sembilan	Kedah	Kelantan		Melaka
		Pulau Pinang			

**Table 2:** Prevalence of bovine fascioliasis cases for eleven-year period from five Regional Veterinary Laboratories in Peninsular Malaysia.

RVL	No. of positive samples	Total number of samples submitted	Occurrence (%)	Odds ratio and 95% confidence interval
Johor Bahru	1	1136	0.09%	NA
Kelantan	0	208	0.00%	NA
Kuantan	15	302	4.96%	1
Bukit Tengah	19	342	5.55%	1.2 (0.6, 2.3)
Sepang	0	0	0.00%	NA
Total	35	1988	1.76%	

## Data Availability

The datasets supporting the conclusions of this article are included within the article. Raw data are available from the authors upon request.
